# A Simple Method for High-Performance, Solution-Processed, Amorphous ZrO_2_ Gate Insulator TFT with a High Concentration Precursor

**DOI:** 10.3390/ma10080972

**Published:** 2017-08-21

**Authors:** Wei Cai, Zhennan Zhu, Jinglin Wei, Zhiqiang Fang, Honglong Ning, Zeke Zheng, Shangxiong Zhou, Rihui Yao, Junbiao Peng, Xubing Lu

**Affiliations:** 1 Institute of Polymer Optoelectronic Materials and Devices, State Key Laboratory of Luminescent Materials and Devices, South China University of Technology, Guangzhou 510640, China; c.w01@mail.scut.edu.cn (W.C.); mszhuzn@mail.scut.edu.cn (Z.Z.); 201620108095@mail.scut.edu.cn (J.W.); fangzq1230@gmail.com (Z.F.); 201520114219@mail.scut.edu.cn (Z.Z.); earth7788@163.com (S.Z.); psjbpeng@scut.edu.cn (J.P.); 2 Institute for Advanced Materials and Guangdong Provincial Key Laboratory of Quantum Engineering and Quantum Materials, South China Normal University, Guangzhou 510006, China; luxubing@scnu.edu.cn

**Keywords:** solution-processed ZrO_2_, low leakage current density, precursor concentration, control thickness

## Abstract

Solution-processed high-k dielectric TFTs attract much attention since they cost relatively little and have a simple fabrication process. However, it is still a challenge to reduce the leakage of the current density of solution-processed dielectric TFTs. Here, a simple solution method is presented towards enhanced performance of ZrO_2_ films by intentionally increasing the concentration of precursor. The ZrO_2_ films not only exhibit a low leakage current density of 10^−6^ A/cm^2^ at 10 V and a breakdown field of 2.5 MV/cm, but also demonstrate a saturation mobility of 12.6 cm^2^·V^−1^·s^−1^ and a I_on_/I_off_ ratio of 10^6^ in DC pulse sputtering IGZO-TFTs based on these films. Moreover, the underlying mechanism of influence of precursor concentration on film formation is presented. Higher concentration precursor results in a thicker film within same coating times with reduced ZrO_2_/IGZO interface defects and roughness. It shows the importance of thickness, roughness, and annealing temperature in solution-processed dielectric oxide TFT and provides an approach to precisely control solution-processed oxide films thickness.

## 1. Introduction

The development of high mobility, thin film transistors (TFTs) depends heavily on the selection of dielectric materials with appropriate characteristics, including low leakage current and high breakdown voltage [1,2]. High-k dielectrics have been widely employed into facilitating TFT due to its high capacitance, which is beneficial to a large I_on_/I_off_ ratio and low-voltage operation. Although the vacuum-based deposition methods have their own advantages, their high fabrication cost restricts the areas of their applications [3,4]. Deposition with solution-based approaches has been reported as an alternative for a few material systems, including HfO_2_, ZrO_2_, and Y_2_O_3_ [5]. Solution-processed gate dielectrics are required to have a low leakage current (<10^−5^ A/cm^2^) and a smooth surface (RMS < 1 nm), which is beneficial to a stable operating current and a high I_on_/I_off_ ratio larger than 10^6^. Also, the breakdown electric field is necessary to those larger than 2 MV/cm to satisfy most operating conditions of the TFT device [3]. However, in most of the previous reports, solution-processed dielectric TFTs have had a large leakage current density under low operating voltage with a relatively low breakdown field [6].

Several methods [7,8,9,10] towards reducing the leakage current are mentioned in the previous study including doping, applying the modified layer, and increasing the annealing temperature. Most of them are related to a complicated operation in regulation of the precursor and interface engineering. Increasing the annealing temperature is an effective way to enhance the device electric performance, which is more convenient compared to other methods. It is noted that oxide dielectric will crystallize at a high annealing temperature, and the grain boundaries created by crystallization provide pathways for the leakage current, resulting in a worse device performance [11]. The balance between increasing annealing temperature and maintaining the amorphous state remains an issue for the solution-processed oxide dielectric. Besides that, increasing thickness is a simple way [12], but it is hard to precisely control the thickness of solution-processed films by adjusting the processing parameter or increasing the coating times due to complicated fluid property [13].

## 2. Results and Discussion

**Figure 1:**
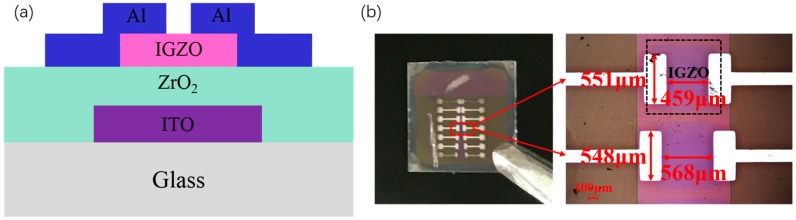
(**a**) The device structure of solution-processed, ZrO_2_-based TFT; (**b**) picture of solution-processed ZrO_2_ TFT.

In this work, a simple strategy is proposed to improve the electrical performance of the solution-processed ZrO_2_ gate insulator TFTs by increasing the concentration of the precursor. A metal-insulator-metal (MIM) capacitor structure of ITO/ZrO_2_/Al is prepared to characterize the ZrO_2_ dielectric layer. Also, the structure of the solution-processed ZrO_2_ TFT is shown in Figure 1.

**Figure 2:**
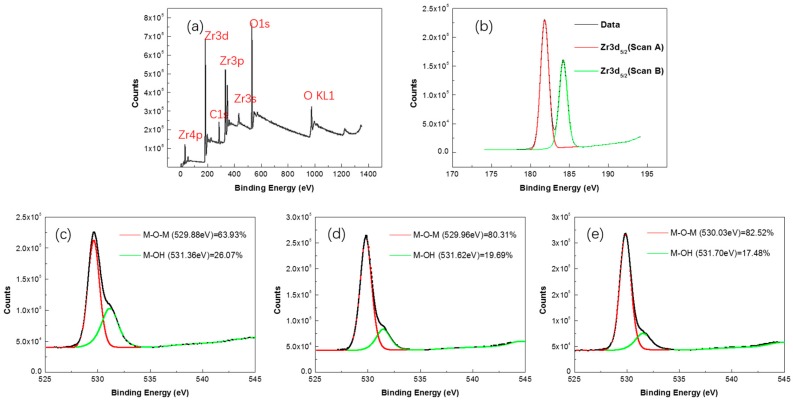
(**a**) X-ray photoelectron spectroscopy (XPS) spectra of ZrO_2_ films annealed at 300 °C; (**b**) Zr 3d spectra of ZrO_2_ films annealed at 300 °C; (**c**–**e**) O 1s spectra of ZrO_2_ films annealed at 300 °C, 400 °C, and 500 °C, respectively.

The annealing process for solution-processed oxide dielectric is significant since during the annealing process an unnecessary constituent is removed and oxide is developed. As is shown in Figure 2a, ZrO_2_ film annealed at 300 °C shows obvious zirconium and an oxygen peak but not obvious chlorine. Since the solution from the precursor is expected to have carbon or chlorine, the absence of Cl indicates the removal of the solvent, followed by densification and a reaction to form the ZrO_2_ film. Furthermore, the atomic ratio of Zr to O is calculated to be around 1:2 after calibration of all peaks [14,15], indicating that the deposited film is oxidized to form ZrO_2_, as expected (Figure 2b). In Figure 2c–e, we fitted the oxygen 1s peak to a superposition of two peak components. The peaks centered at 530.0 ± 0.2 eV and 531.5 ± 0.2 can be assigned to the metal-oxygen-metal M-O-M species and M-OH species [8,16] in both films annealed at 300 °C, 400 °C, and 500 °C, respectively. The M-O-M species (530.0 ± 0.2 eV) is related to O^2−^ ions combined with the Zr^4+^ ion, while the M-OH species (531.5 ± 0.2 eV) is attributed to oxygen defects in the film and the presence of loosely bonded oxygen molecules such as adsorbed O_2_ and H_2_O. The percentage of the M-O-M species reflects a combination of metal and oxygen after the annealing process, while the M-OH species is related to a defect state in the films. In other words, higher the percentage of the M-O-M species in the O 1s XPS spectrum results in a denser film with a lower defect state, which is good for the electric performance of the gate insulator as it decreases access for leakage current and improves the on/off ratio for the TFT device [17]. Although both samples contain an appreciable concentration of M-OH defects, we can clearly see that the concentration of the M-O-M species increases when the annealing temperature increases from 300 °C to 500 °C. This reveals that the sintering of the precursor into a dense oxide film is more sufficient with the increasing of the annealing temperature.

**Figure 3:**
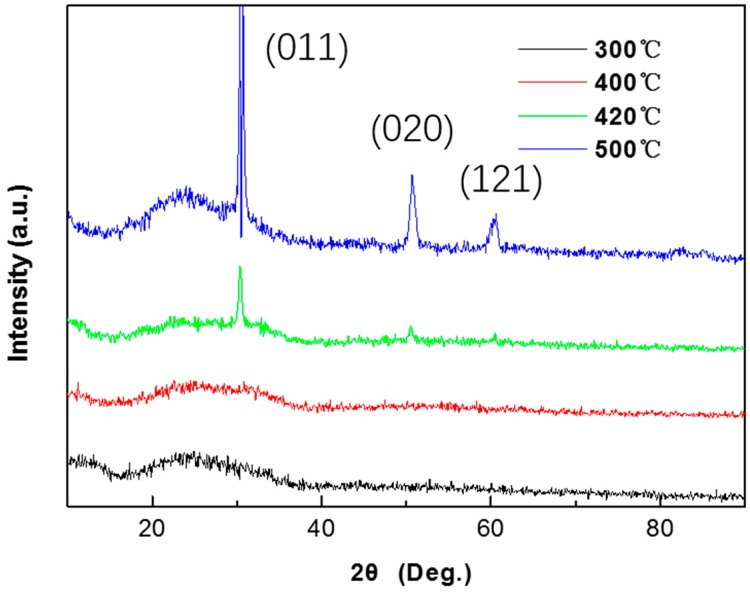
X-ray diffraction (XRD) scans obtained from ZrO_2_ film coated on the glass of a different annealing temperature.

However, oxide will crystallize at a high annealing temperature, which is bad for the electrical performance of dielectric films. Obvious diffraction peaks are observed at 30.2°, 51.6°, and 60.1° in the ZrO_2_ layer annealed above 420 °C, as shown in Figure 3, which correspond to the (011), (020), and (121) planes [18]. Crystallization in dielectric films can provide pathways for a leakage current due to the grain boundaries, leading to a large leakage current and a small on/off ratio for the TFT device. When ZrO_2_ film heats up to 420 °C it starts to crystallize, so the electrical performance turns bad for devices annealed over 420 °C. This is contradictory to the conclusion in the previous paragraph which pointed out that high annealing temperature results in denser films and a lower leakage current density, and for dielectric film, the amorphous state and high annealing temperature is beneficial but the critical factor is the crystallization of ZrO_2_. So, here we choose 400 °C as the post annealing temperature, while the ZrO_2_ films get adequate heat treatment and stay in an amorphous state at the same time.

**Figure 4:**
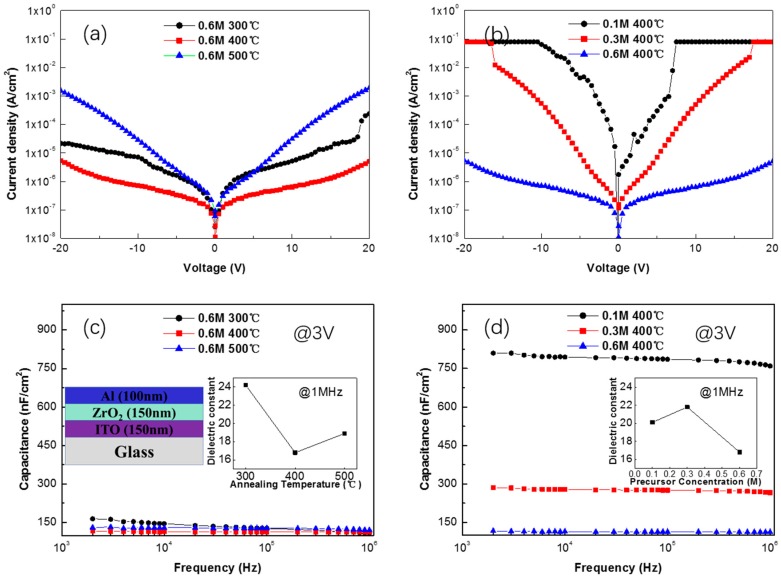
Leakage current vs applied voltage for (**a**) different annealing temperature and (**b**) different concentration precursor-based ZrO_2_ film; areal capacitance vs frequency for (**c**) different annealing temperature and (**d**) different concentration precursor-based ZrO_2_ film.

As shown in Figure 4a, the leakage current density and capacitance of ZrO_2_ film fabricated by the 0.6 M precursor with a different annealing temperature is consistent with the assumption above. When the annealing temperature increases from 300 °C to 500 °C, leakage current density for the annealing temperature of 300 °C, 400 °C, and 500 °C, measured at an applied voltage of 10 V, firstly decreases as the annealing temperature raises from 300 °C to 400 °C, and then increases when the annealing temperature reaches 500 °C. It is known that the ZrO_2_ films in the experiment crystallize at 420 °C, so when the annealing temperature increases from 300 °C to 400 °C, the ZrO_2_ films still remain in an amorphous state and attain a denser film with a higher percentage of M-O-M species and less residual components. Both MIM devices fabricated by the 0.6 M precursor show a breakdown voltage higher than 30 V (about 2.5 MV/cm, not shown in the figure). The ZrO_2_ film fabricated by the 0.6 M precursor annealed at 400 °C exhibits a best performance in our experiment of leakage current density lower than 10^−6^ A/cm^2^ at 10 V and just 10^−5^ A/cm^2^ at 20 V. The capacitance for 0.6 M ZrO_2_ film is around 125 nF/cm^2^ at a frequency of 1 MHz, has little relation with annealing temperature, and is stable along with the increase of frequency [19]. We also prepare the ZrO_2_ film from a different concentration precursor. Both 0.1 M, 0.3 M, and 0.6 M ZrO_2_ film are spin coated three times. As shown in Figure 4b, ZrO_2_ films prepared from 0.1 M and 0.3 M precursor show a relatively high leakage current density as the precursor concentration decreases, and have a breakdown voltage between 10 V and 15 V. The capacitance for 0.1 M and 0.3 M ZrO_2_ films is 760 nF/cm^2^ and 280 nF/cm^2^. Regarding their thickness, the dielectric constant of different films is calculated to be 15–22 for both films of a different condition, while the small fluctuation of capacitance of the different films can be ascribed to errors in the measurements [20,21].

**Figure 5:**
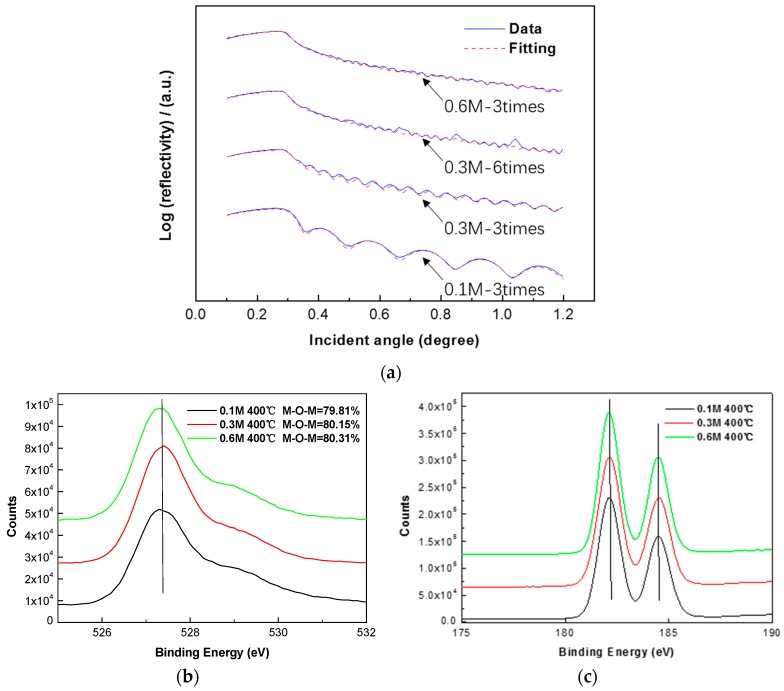
(**a**) X-ray reflectivity measurements of 0.1 M, 0.3 M, and 0.6 M precursor-based ZrO_2_ film of different coating times; (**b**) O 1s spectra; (**c**) Zr 3d spectra of ZrO_2_ films from 0.1 M, 0.3 M, and 0.6 M precursor.

To find out the reason for the leakage current density difference, we investigate the thickness and elementary composition of films from the different concentration precursor. The thickness of 0.1 M, 0.3 M, and 0.6 M ZrO_2_ with coating times of 3 are 22.5 ± 0.5 nm, 66.9 ± 0.5 nm, and 137.1 ± 0.5 nm, respectively, which is shown in Figure 5. The thickness of the ZrO_2_ film is nearly proportional to the concentration of the precursor used for spin-coating. No shift is observed in Figure 5b,c of the position of M-O-M peak and the Zr 3d peaks. The M-O-M species remains 80% ± 0.2% of both ZrO_2_ films from the 0.1 M, 0.3 M, and 0.6 M precursors. The XPS results explain that the improvement of the leakage current density of 0.6 M ZrO_2_ films to 0.3 M and 0.1 M films is irrelevant to changes in composition. This can well explain the leakage current density difference, as a high concentration precursor results in thicker films within the same coating times, which effectively impedes electron transportation from gate electrode to source/drain electrodes. Furthermore, when we spin coat 0.3 M ZrO_2_ film six times, it shows a thickness of 129.3 ± 0.5 nm. Also, the thickness of the ZrO_2_ film is proportional to the coating times as well. The density of the ZrO_2_ film seems to be irrelevant to the concentration or thickness, while the roughness increases with thickness or coating times. We also prepare a ZrO_2_ film from a ≥0.7 M precursor, and cracks occur after annealing at 300 °C. The solubility of ZrOCl_2_·8H_2_O depends on solvent, and an inorganic chemistry solvent such as 2-methoxyethanol or ethylene glycol is a better choice than acetylacetone, which might dissolve no more than 0.1 M Zr of matrix material [11]. The data obtained from XRR of 0.1 M, 0.3 M, and 0.6 M ZrO_2_ films of different coating times are summarized in Table 1.

The theoretical simulations curves are in good agreement with the realistic measurement curves for the films coated three times, but are a little misaligned for the films coated six times. This is also reflected in the roughness of the films. The films with coating times of six exhibit a higher surface roughness than those of three times, which might damage the thin IGZO semiconductor fabricated on the top of the dielectric layer, resulting in an unstable operating current and a lower I_on_/I_off_ ratio. We can draw a conclusion that although thickness is the key factor in reducing the leakage current in ZrO_2_ TFT, it is not suitable to obtain thick films by increasing coating times excessively, in consideration of the interface roughness between the dielectric layer and the semiconductor.

**Figure 6:**
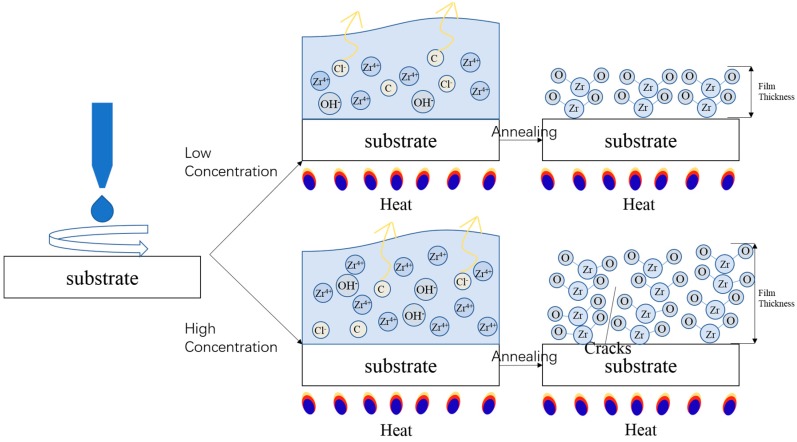
Effect of precursor concentration of film formation.

The film formation process is described in Figure 6. After the precursor is spin-coated on the substrate, a solution layer is formed containing a large number of ions [6]. The solution layer has a certain thickness which has little dependency on the solution viscosity, surface tension, and concentration, because in the spin-coating process centrifugal force plays a dominant role. The solution layer has a larger thickness than the final ZrO_2_ film after post-annealing, and the high concentration precursor results in more ions in this thick layer. During the annealing process water evaporates, accompanied by the removal of the Cl and C elements in the solution layer [22]. The Zr and O elements move in the solution and recombine to form the Zr-O bonding. Finally, ZrO_2_ pile up in good order to form films with certain thicknesses, which are related to the quantity of Zr ions in the precursor. As for the situation in which precursor concentration is very high (≥0.7 M in 10 mL 2-MOE), an orienting, ordered arrangement cannot well develop before the liquid totally evaporates, and regions with disordered orientation might generate too much stress during the annealing process [23,24], leading to cracks growing and, finally, the fracture of these films. Similarly, the high concentration precursor (0.3 M–0.6 M) leads to high surface roughness due to the irregular arrangement of atoms, which might also happen within too many coating times. This model of the film formation process can well explain the phenomenon in the spin-coating oxide precursor, and points out that we can precisely control the film thickness of the solution-processed oxide by adjusting the precursor concentration.

**Figure 7:**
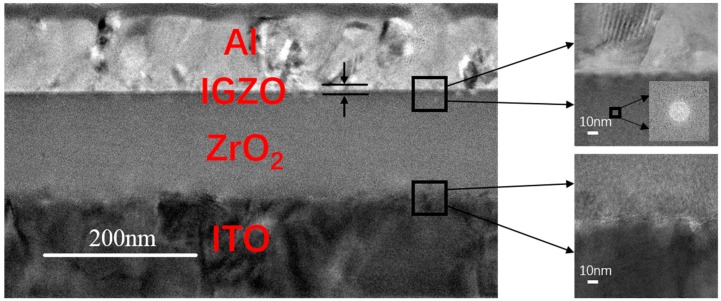
The cross-sectional high-resolution transmission microscopy (HRTEM) images of 0.6 M ZrO_2_-TFT.

**Figure 8:**
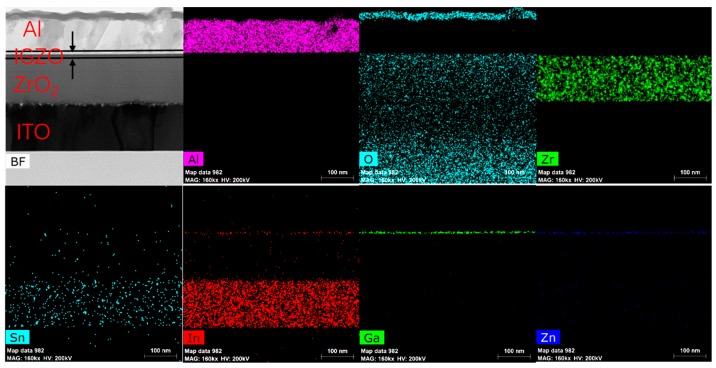
Energy dispersive X-ray spectrometer (EDS) of cross-sectional 0.6 M ZrO_2_-TFT.

As shown in Figure 7, solution-processed ZrO_2_ from the 0.6 M precursor annealed at 400 °C is very dense and has good contact with both the IGZO and ITO bottom gate. The interface of IGZO/ZrO_2_ is smooth, resulting in a good transportation of carriers [25]. Also, holes exist in the interface of ZrO_2_/ITO due to the deposition process of the ITO bottom gate, which has little effect on the electric performance of the TFT device [26]. As shown in the figure, some crystalline features with a distinct atomic arrangement in both Al and ITO are observed, indicating that Al and ITO are a single-crystal grow in ZrO_2_-TFT. However, ZrO_2_ film stays in an amorphous state, which is consistent with the XRD diffraction pattern. In Figure 8, a clear boundary of each layer is observed, as In, Ga, and Zn stand for the IGZO layer. No diffusion of Al into IGZO or segregation of IGZO is observed in EDS mapping, and oxygen uniformly distributes in the IGZO, ZrO_2_, and ITO films. The image of IGZO/ZrO_2_ interface demonstrates the good coverage ability of the spin-coating ZrO_2_ film, since the contact between IGZO/ZrO_2_ is very tight. The HRTEM images and EDS mapping sufficiently prove that the solution-processed ZrO_2_ film obtained from the 0.6 M precursor is smooth, dense, and in the amorphous state as well.

**Figure 9:**
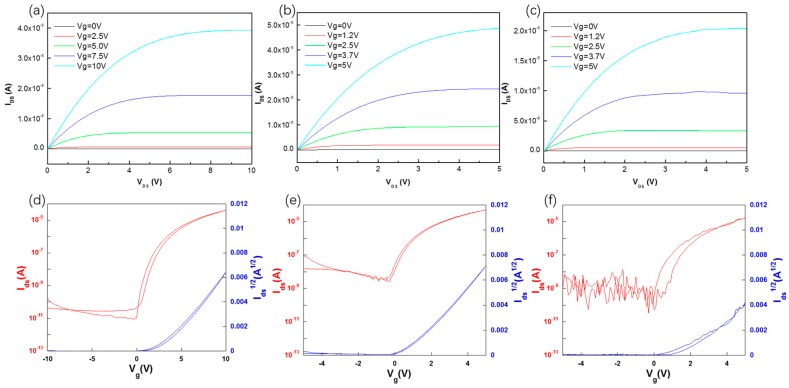
Output characteristics of (**a**) 0.6 M ZrO_2_-TFT coated three times; (**b**) 0.3 M ZrO_2_-TFT coated three; and (**c**) 0.3 M ZrO_2_-TFT coated six times; transfer characteristics of (**d**) 0.6 M ZrO_2_-TFT coated three times; (**e**) 0.3 M ZrO_2_-TFT coated for three; and (**f**) 0.3 M ZrO_2_-TFT coated six times.

Figure 9a–c shows the representative output characteristics of ZrO_2_-TFTs and (d–f) shows the transfer characteristics; both devices exhibit a clear pinch-off and relatively low threshold voltage, which benefit from the low leakage current and large band gap of ZrO_2_ [27]. Attaining operating conditions under low voltage is attributed to large dielectric constant relative to other dielectrics. As can be seen in Figure 9d, ZrO_2_-TFT fabricated from the 0.6 M ZrO_2_ precursor, annealed at 400 °C, has an improvement in the I_off_ and I_on_/I_off_ ratios compared to the TFT from the 0.3 M ZrO_2_ precursor, owing to low leakage current density at off state. According to the results from the XRD and XPS analyses, 0.6 M ZrO_2_ annealed at 400 °C has a higher percentage of metal-oxide bonding, and maintaining an amorphous state at the same time leads to a denser ZrO_2_ film with fewer oxygen defects and grain boundaries which might serve as pathways for leakage current [28]. Although the 0.3 M ZrO_2_-TFT coated six times and annealed at 400 °C has a similar thickness to the 0.6 M ZrO_2_-TFT, it shows an unstable current at the negative voltage region owing to the relative large roughness at the interface of ZrO_2_/IGZO. So, devices from the 0.6 M ZrO_2_ precursor not only allow the low leakage current but also exhibit low surface roughness, resulting in high mobility of 12.6 cm^2^·V^−1^·s^−1^ and a large I_on_/I_off_ ratio of 10^6^.

## 3. Materials and Methods

The ZrO_2_ solution was synthesized by dissolving 0.1 M, 0.3 M, and 0.6 M ZrOCl_2_·8 H_2_O in 10 mL 2-methoxyethanol (2 MOE) solvent, respectively [11]. The solution was stirred at 500 r/min at room temperature for 2 h, and was aged for at least one day. A sandwich structure of ITO/ZrO_2_/Al is used to measure the leakage current density and capacitance of the ZrO_2_ films. A 150-nm-thick ITO was deposited on glass substrates as a gate electrode by direct current (DC) sputtering and then patterned by the lithographic process [29]. Subsequently, ZrO_2_ films were formed by spin-coating several times to acquire a certain thickness. 10 nm-thick IGZO was then grown by direct current (DC) pulse sputtering with a pressure of 1mTorr (O:Ar = 5%) and patterned by shadow mask, then annealed at 300 °C for 1 h [30]. The IGZO target is composed of the atomic ratio of In:Ga:Zn:O = 1:1:1:4. The channel width (W) and length (L) of the two structures were 550 μm and 450 μm, respectively, thus the W/L ratio is 1.22. Finally, the Al source/drain electrodes with 150-nm-thickness were deposited by direct current (DC) sputtering at room temperature. All layers, except for ZrO_2_, are prepared under 300 °C.

X-ray photoelectron spectroscopy (XPS) performed by ESCALAB250Xi (Thermo Fisher Scientific, Waltham, MA, USA) at a basic pressure of 10^−9^ mbar is used to reveal the elemental composition of the spin-coated ZrO_2_ film annealed at 300 °C, 400 °C, and 500 °C, respectively. The atomic ratio of Zr to O is calculated after calibration of all the peaks, and the oxygen 1s peak is divided into a superposition of two peak components to analyze the effect of the annealing temperature. X-ray diffraction (XRD) (EMPYREAN, PANalytical, Almelo, The Netherlands) is used to investigate the crystalline phase of the ZrO_2_ film fabricated on the glass substrate. The thickness and roughness of the 0.1 M, 0.3 M, and 0.6 M ZrO_2_ films of different coating times is measured by X-ray reflectivity (XRR) using the same equipment. The thickness can be obtained by fitting the interference fringe of the X-ray. A cross-sectional image of high-resolution transmission microscopy (HRTEM) of JEM-2100 F (JEOL, Tokyo, Japan) is carried out to investigate the interface of ZrO_2_ TFT prepared from the 0.6 M precursor equipped with an energy dispersive X-ray spectrometer (EDS) mapping by Bruker (Berlin, Germany), which can reveal the distribution of the elements of each layer. The electrical characteristics of TFTs were measured using a semiconductor parameter analyzer (Agilent4155C, Agilent, Santa Clara, CA, USA) under an ambient atmosphere. Also, the current-voltage (I-V) and capacitance-frequency (C-f) characteristics of the MIM capacitor were measured by the keithley4200 (Tektronix, Beaverton, OR, USA) parameter analyzer under an ambient condition.

## 4. Conclusions

A solution strategy towards the precise thickness and enhanced performance of the solution-processed ZrO_2_-TFT is proposed by adjusting the concentration of the precursor. The underlying mechanism for the enhanced performance is that the high concentration precursor can attain a relatively thick ZrO_2_ dielectric within fewer coating times, which leads to low leakage current density and a smooth surface for coating films. XPS analysis shows the advantage of high temperature annealing as the percentage of M-O-M species increases from 63.9% to 82.5%, with the annealing temperature increasing from 300 °C to 500 °C. XRD analysis and the IV test reveal crystallization damage of the electric performance for the ZrO_2_ dielectric films, since leakage current density increases by two orders of magnitude when the ZrO_2_ films begin to crystallize. We find a method of the film formation process for the solution-process dielectric films, which explains the effect of different concentration precursor on film property. Film thickness is proportional to precursor concentration under 0.6 M, and surface roughness decreases with a reduction in coating times. As a result, a IGZO-TFT based on 0.6 M solution-processed ZrO_2_ with a thickness of 130 nm and a roughness of 0.79 nm exhibit a low leakage current density of 10^−6^ A/cm^2^ at 10 V and a saturation mobility of 12.6 cm^2^·V^−1^·s^−1^, as well as an I_on_/I_off_ ratio of 10^6^. The image of HRTEM also confirms the smoothness and the amorphous state of the dielectric film fabricated by the 0.6 M ZrO_2_ precursor, which is attributed to a dense film and an outstanding interface between ZrO_2_/IGZO with fewer defect states. This paper offers a simple method by increasing the solution concentration to achieve high mobility solution-processed dielectric TFTs and provide an approach to fabricate the solution-processed oxide dielectric film with precise thickness and a smooth surface.

## Figures and Tables

**Table 1:** The summary of density, thickness, and roughness of 0.1 M, 0.3 M, and 0.6 M precursor-based ZrO_2_ film of different coating times.

Concentration	Layer	Density (g/cm^3^)	Thickness (nm)	Roughness (nm)
0.1 M	3	4.69	22.49	0.51
0.3 M	3	4.83	66.98	0.56
0.3 M	6	4.77	129.29	1.21
0.6 M	3	4.75	137.14	0.79
